# Prognostic Value of Non-Invasive Fibrosis and Steatosis Tools, Hepatic Venous Pressure Gradient (HVPG) and Histology in Nonalcoholic Steatohepatitis

**DOI:** 10.1371/journal.pone.0128774

**Published:** 2015-06-17

**Authors:** Giada Sebastiani, Rasha Alshaalan, Philip Wong, Maria Rubino, Ayat Salman, Peter Metrakos, Marc Deschenes, Peter Ghali

**Affiliations:** 1 Department of Medicine, McGill University Health Centre, Montreal, Quebec, Canada; 2 Section of Hepatobiliary and Transplant Surgery, McGill University Health Centre, Montreal, Quebec, Canada; Taipei Veterans General Hospital, TAIWAN

## Abstract

**Background & Aims:**

Non-invasive diagnostic methods for liver fibrosis predict clinical outcomes in viral hepatitis and nonalcoholic fatty liver disease (NAFLD). We specifically evaluated prognostic value of non-invasive fibrosis methods in nonalcoholic steatohepatitis (NASH) against hepatic venous pressure gradient (HVPG) and liver histology.

**Methods:**

This was a retrospective cohort study of 148 consecutive patients who met the following criteria: transjugular liver biopsy with HVPG measurement; biopsy-proven NASH; absence of decompensation; AST-to-Platelets Ratio Index (APRI), fibrosis-4 (FIB-4), NAFLD fibrosis score, ultrasound, hepatic steatosis index and Xenon-133 scan available within 6 months from biopsy; a minimum follow-up of 1 year. Outcomes were defined by death, liver transplantation, cirrhosis complications. Kaplan–Meier and Cox regression analyses were employed to estimate incidence and predictors of outcomes, respectively. Prognostic value was expressed as area under the curve (AUC).

**Results:**

During a median follow-up of 5 years (interquartile range 3-8), 16.2% developed outcomes, including 7.4% who died or underwent liver transplantation. After adjustment for age, sex, diabetes, the following fibrosis tools predicted outcomes: HVPG >10mmHg (HR=9.60; 95% confidence interval [CI] 3.07-30.12), histologic fibrosis F3-F4 (HR=3.14; 1.41-6.95), APRI >1.5 (HR=5.02; 1.6-15.7), FIB-4 >3.25 (HR=6.33; 1.98-20.2), NAFLD fibrosis score >0.676 (HR=11.9; 3.79-37.4). Prognostic value was as follows: histologic fibrosis stage, AUC=0.85 (95% CI 0.76-0.93); HVPG, AUC=0.81 (0.70-0.91); APRI, AUC=0.89 (0.82-0.96); FIB-4, AUC=0.89 (0.83-0.95); NAFLD fibrosis score, AUC=0.79 (0.69-0.91). Neither histologic steatosis nor non-invasive steatosis methods predicted outcomes (AUC<0.50).

**Conclusions:**

Non-invasive methods for liver fibrosis predict outcomes of patients with NASH. They could be used for serial monitoring, risk stratification and targeted interventions.

## Introduction

Nonalcoholic fatty liver disease (NAFLD) is a disease spectrum ranging from simple steatosis to nonalcoholic steatohepatitis (NASH), which is a state of hepatocellular inflammation and damage in response to the accumulated fat within liver parenchyma [1]. An estimated 20–46% of North American adults, approximately 90 million people, have NAFLD [2,3]. NASH could be present in one third of NAFLD cases [4]. Currently, NASH represents the second or third most common indication for liver transplantation in North America and it has been projected to become the leading indication over the next 10 to 20 years [5,6]. Approximately 20–40% of patients with NASH will develop significant liver fibrosis, 10% will progress to cirrhosis and 1–5% will develop hepatocellular carcinoma (HCC) [7]. Early identification of patients at risk for worse prognosis is of paramount importance for risk stratification, initiation of cirrhosis surveillance protocols and interventions, optimization of healthcare resources, counseling and referral for liver transplantation [1,8]. Staging of liver fibrosis is pivotal for prognosis of patients with NASH [9]. Patients with fibrosis stage 3–4 have increased overall mortality [10]. A portal pressure gradient >10 mmHg defines clinically significant portal hypertension and it is predictive of hepatic decompensation [8]. End-stage liver complications, including esophageal varices and ascites, mainly occur in patients with advanced liver fibrosis stages and clinically significant portal hypertension [8,11]. The severity of hepatic steatosis may also have clinical implications as it has been associated with higher mortality caused by liver disease and with increased risk of cardiovascular disease and HCC [10,12,13]. Liver biopsy has long been the gold standard to diagnose NASH, stage fibrosis and grade steatosis. However, liver biopsy is unfeasible as serial monitoring tool for prognostication because of its invasiveness, cost, sampling error [9,14]. The measurement of hepatic venous gradient pressure (HVPG) is the gold standard for detection of clinically significant portal hypertension. However, the measurement of HVPG through transjugular catheterization of hepatic vein is also invasive and unfeasible as screening prognostic tool. Non-invasive tools have been proposed to stage liver fibrosis and to predict presence of cirrhosis complications, including serum biomarkers, such as fibrosis-4 (FIB-4), AST-to-Platelet ratio index (APRI), NAFLD fibrosis score, and measurement of liver stiffness by transient elastography (Fibroscan) [15–20]. Non-invasive methods can also diagnose and grade steatosis, including radiologic techniques, such as ultrasonography [21] and Xenon-133 liver scan [22], and biomarkers such as hepatic steatosis index (HSI) [23]. Non-invasive tools for liver fibrosis may help identify patients at high risk for liver-related complications or death in chronic hepatitis C and B, and in NAFLD [24–26]. No study has specifically investigated the prognostic performance of non-invasive methods for liver fibrosis and hepatic steatosis for prediction of clinical outcomes in NASH as compared to the gold standard methods to diagnose liver fibrosis (histology) and clinically significant portal hypertension (HVPG).

The aim of this study was to investigate the prognostic value of non-invasive tools for liver fibrosis and steatosis, HVPG and histology for predicting death and liver-related outcomes in patients with NASH.

## Patients and Methods

### Study design and population

This was retrospective cohort study conducted at a single site, the Division of Gastroenterology and Hepatology of the Royal Victoria Hospital, McGill University Health Centre. All eligible consecutive patients with histological diagnosis of NASH and in active follow-up were included. In order to be included, patients had to fulfill the following criteria: (a) age > 18 years; (b) transjugular liver biopsy with measurement of HVPG; (c) biopsy-proven diagnosis of NASH; (d) absence of hepatic decompensation and HCC at entry; (e) availability within 6 months from liver biopsy of APRI, FIB-4, NAFLD fibrosis score, ultrasound, hepatic steatosis index and Xenon-133 scan; (f) at least 2 study visits between 2004–2013; (g) a minimum follow-up of 1 year. Exclusion criteria were: (a) positivity for HCV antibody and/or hepatitis B surface antigen; (b) significant alcohol intake, defined as per guidelines of the American Association for the Study of Liver Diseases (1); (c) liver transplantation; (d) last follow up visit antecedent to January 2011; (e) length of liver biopsy specimen < 1cm. Patients were followed until January 2014 or were censored either when they died or at their last clinic visit.

### Ethics statement

At the time of liver biopsy procedure, all the participants provided informed written consent to participate in the study. The Institutional Research Ethic Board of the Research Institute of McGill University Health Center approved the study, which was conducted according to the Declaration of Helsinki.

### Clinical and biological parameters

Clinical parameters included age, gender, body mass index (BMI), history of diabetes, hypertension or dyslipidemia, history of hepatic decompensation, HCC or liver transplantation. BMI was categorized using the World Health Organization international classification system [27]. Obesity was defined as BMI >30 Kg/m2. Metabolic syndrome was defined according to the criteria of the International Diabetes Federation [28]. Biological parameters included platelets, AST, ALT, albumin, total cholesterol, fasting glucose. The following simple biomarkers for liver fibrosis were calculated: APRI defined as: [100 x (AST/upper limit of normality)/platelet count (10^9^/L) [15]; FIB-4 calculated as: age (years) × AST /platelet count (10^9^/L)]× ALT½ (16); NAFLD fibrosis score defined as: -1.675 + 0.037 × age (years) + 0.094 × BMI + 1.13 × diabetes + 0.99 × AST/ALT]– 0.013 × platelet count 10^9^/L)– 0.66 × albumin (g/dl) [17]. HSI was calculated as follows: 8 × AST/ALT + BMI (+2, if female; +2, if diabetes) [23]. Abdominal ultrasound was performed by two experienced radiologists at the Department of Radiology of the McGill University Health Centre. Hepatic steatosis was diagnosed on the basis of characteristic imaging findings, namely bright liver pattern, liver-kidney contrast, vascular blurring and/or deep hepatic attenuation. Xenon-133 liver scan was performed as previously described [22]. Hepatic steatosis was graded from 0 to 3: grade 0 no radioactivity in the hepatic area at any time, while grade 3 corresponds to radioactivity comparable with the greatest activity in the lung.

### Histological Assessment

The main indication for liver biopsy was confirmation of clinical suspicion of nonalcoholic steatohepatitis (NASH): ultrasonographic steatosis with presence of metabolic syndrome or elevated transaminases with any among obesity, diabetes, dyslipidemia. In a minority of cases (<10%), a liver biopsy was requested for suspected comorbidity or overlap diagnosis (autoimmune hepatitis, cholestatic disease, hemochromatosis). Based on emerging data from the literature, the transjugular approach has been the preferred method for the diagnosis of NASH in our center since 2008. Indeed, histologic lesions of NASH are unevenly distributed throughout the liver parenchyma. Studies comparing two percutaneous liver biopsy samples from NAFLD patients observed that while the consistency in fatty change is relatively high (78%), the fibrosis stage was different between the two samples in 41% of cases [29]. Moreover, HVPG is regarded as the standard of reference for diagnosis of portal hypertension and for an accurate classification of the spectrum of patients with advanced liver fibrosis/cirrhosis [8]. Transjugular liver biopsy has become the preferred method for diagnosis and staging of NASH in our institution given the risk of underestimation of liver fibrosis stage, which could be counterbalanced by the adjunctive value of HVPG.

All liver biopsies were interpreted by a single experienced liver pathologist. The stage of steatosis and fibrosis were reported according to the Brunt classification [30]. Briefly, fibrosis was staged as follows: stage 0—no fibrosis; stage 1—portal fibrosis without septa; stage 2—portal fibrosis with few septa; stage 3—numerous septa without cirrhosis; stage 4—cirrhosis. The degree of fatty infiltration was assessed and graded based on percentage of involved hepatocytes: mild (up to 33%); moderate (33% to 66%); and severe (>66%). In 95 patients (64.2%), NAFLD activity score (NAS) was also available. NAS was calculated as the unweighted sum of the scores for steatosis (0–3), lobular inflammation (0–3) and hepatocellular ballooning (0–2) [31].

### Measurement of HVPG

All patients underwent measurement of HVPG under local anesthesia; a venous introducer was placed in the right internal jugular vein by the Seldinger technique. A 7F balloon-tipped catheter (Medi-Tech Boston Scientific Cork, Cork, Ireland) was guided into the right hepatic vein for measurement of wedged and free hepatic venous pressure. Adequacy of occlusion was checked by injection of 5 mL of iodinated radiological contrast medium (Iopamiro 370; Bracco, Milano, Italy). The HVPG was calculated as the difference between wedged hepatic venous pressure and free hepatic venous pressure. All measurements were performed in triplicate and a permanent tracing was obtained on a multichannel recorder. A HVPG >10mmHg identified clinically significant portal hypertension [8]. The coefficient of variation in measurements of HVPG was consistently <4%.

### Transient elastography examination

Because the study period started in 2004, transient elastography was available only for a subgroup of patients. In fact, the first transient elastography machine was available in our institution as of 2010. The examination was performed as per manufacturer recommendation, by the same experienced operator. Significant liver fibrosis was diagnosed when liver stiffness was >8kPa [19].

### Primary outcome analysis and exposure measures

Patients were divided into two fibrosis or steatosis risk groups (low, high) for each non-invasive tool according to cut-off values described in the original publications. The following cut-off values were applied to classify patients in the high fibrosis risk group: APRI >1.5; FIB-4 >3.25; NAFLD fibrosis score >0.676. The following cut-off values were used to classify patients in the high steatosis risk group: HSI >36; ultrasound indicating severe steatosis; Xenon scan indicating grade 3 steatosis. Patients were also classified in high risk category on the basis of baseline histology and HVPG, as follows: fibrosis stage >3; steatosis grade = 3; HVPG >10mmHg. Clinical outcomes recorded during the follow-up period were death, liver transplantation and end-stage hepatic complications defined as HCC, ascites, spontaneous bacterial peritonitis, hepatic encephalopathy, de novo varices or significant worsening of varices (such as bleeding or high-risk stigmata on endoscopy).

### Follow-up

The follow-up period ended on January 30^th^, 2014. Patients were censored on their last clinic visit prior to this date or when an outcome occurred. During this period, patients were followed at varied intervals, ranging from 3 to 18 months. At each visit, complete medical history, history of alcohol consumption and physical examination were performed along with routine laboratory work-up to follow their liver disease. A conventional treatment of the underlying liver disease and surveillance was offered during the follow-up. Patients were all counseled about achieving and maintaining appropriate body weight with increased physical activity and diet change.

### Statistical analysis

Baseline (time zero) corresponded to the day of transjugular liver biopsy. We compared characteristics of patients who developed outcomes with those who did not at baseline using T-test for continuous variables and Pearson’s **χ**² or Fisher's exact test for categorical variables. All tests were two-tailed and with a significance level of **α** = 0.05. We estimated incidence rates of outcomes by dividing the number of participants developing the outcome by the number of person-years (PY) of follow-up. Poisson count models were used to calculate confidence intervals for incidence rates. Kaplan-Meier curves were used to illustrate the cumulative incidence of clinical outcomes in patients in the high risk and low risk category. The log-rank test was used to compare incidences among fibrosis and steatosis risk groups for each non-invasive test. Multivariate Cox regression models were built to assess the effect of fibrosis and steatosis risk group on the development of clinical outcomes during follow-up. Final models were adjusted for covariates that were not included already in the biomarkers, namely age, sex, diabetes. We considered an association with the outcome significant when the 95% confidence interval (CI) excluded one. The performance of prognostic tools to predict clinical outcomes was measured as area under the receiver operating characteristic curve (AUC). Standard errors of AUC were calculated by DeLong method. Statistical analysis was performed using STATA 13.1.

## Results

**Fig 1:**
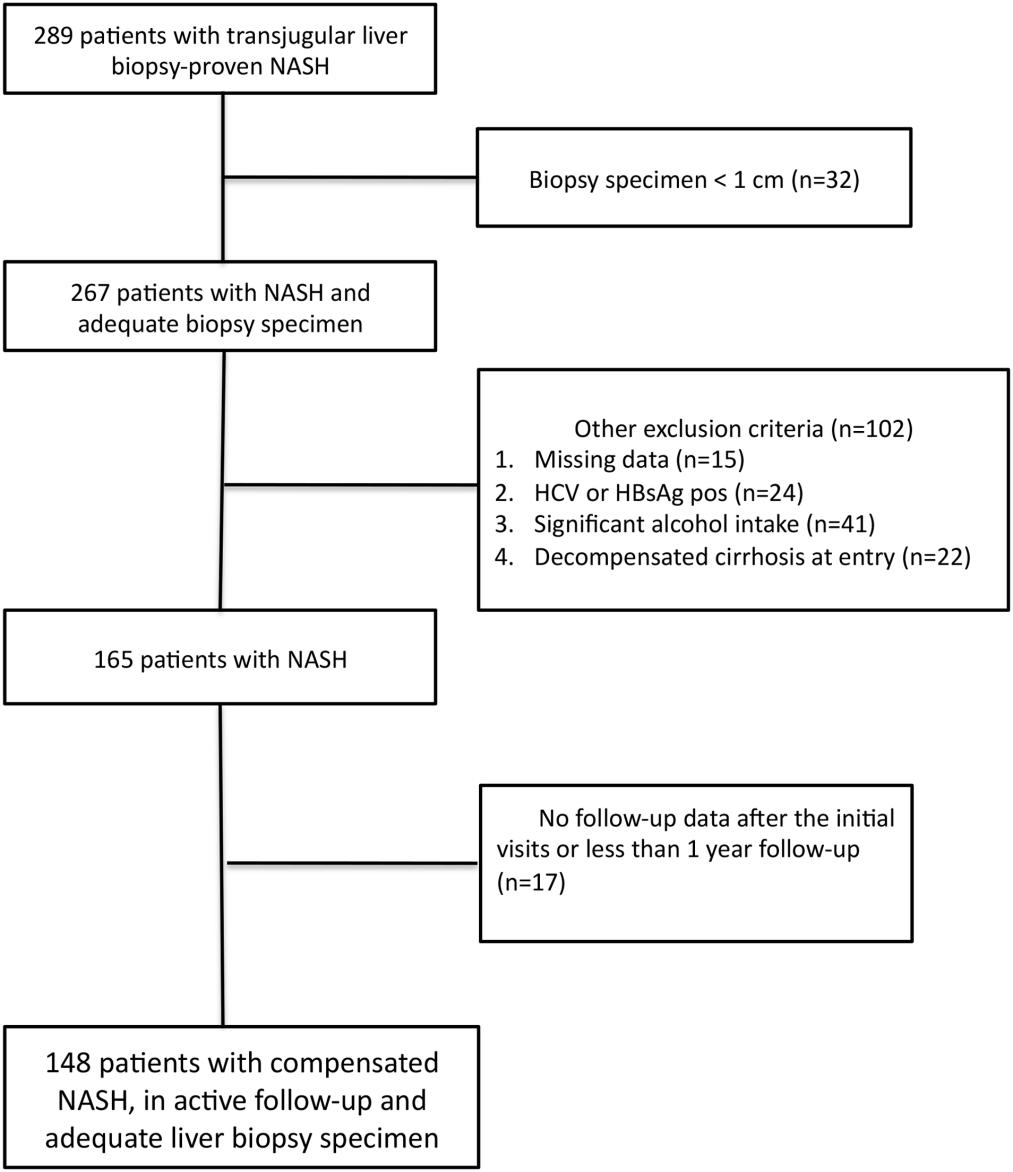
Flow chart displaying selection of study participants for analysis. Out of 289 patients with biopsy proven NASH by transjugular liver biopsy, 32 were excluded for inadequate biopsy specimen. After further exclusion of patients with missing data, those with anti-HCV or HBsAg positivity, patients with significant alcohol intake, the ones with decompensated cirrhosis at entry and patients with insufficient follow-up, the remaining sample of 148 consecutive patients with NASH was included in the present study.

After applying exclusion criteria (Fig 1), 148 patients with NASH and at least two follow-up visits were included. The main clinical, biochemical and histological characteristics of the study population by clinical outcomes status are summarized in Table 1. Overall, there were 103 males and mean age was 49.6 years. The mean BMI was 31.3Kg/m^2^, 74 (56.1%) patients were obese. The mean length of the liver specimen was 1.7cm (standard deviation, [SD] 0.4). Advanced liver fibrosis (F3 or F4 by Brunt) was present in 33.8% of cases. Clinically significant portal hypertension (HVPG >10mmHg), was present in 27 (18.2%) cases. Patients who developed clinical outcomes during the follow-up period had higher prevalence of diabetes, lower platelets, higher bilirubin, lower albumin and higher glucose at baseline. Moreover, patients who developed clinical outcomes had more frequently advanced histologic liver fibrosis and higher HVPG at baseline. Serum fibrosis biomarkers at baseline were significantly higher in patients who developed clinical outcomes than those who did not. Out of 95 patients with available NAS, 75 (79%) had a score of 0–4 and 20 (21%) had a score of 5–8. There was no significant difference in baseline NAS between patients who developed the outcomes and those who did not (p >0.05).

**Table 1:** Demographic, clinical and histological characteristics of 148 patients with NASH at baseline and comparison of those with and without clinical outcomes.

Variable	Whole cohort (n = 148)	Patients with clinical outcomes (n = 24)	Patients without clinical outcomes (n = 124)	p
**Age** (years)	49.5+10.5	53.3+7.9	49.2+29.7	0.07
**Male sex** (%)	103 (69.6)	16 (66.7)	87 (70.2)	0.73
**BMI** (Kg/m^2^)	31.3+5.4	31.7+5.3	31.2+5.4	0.38
**Diabetes** (%)	49 (33.1)	15 (62.5)	34 (27.4)	<0.001
**Hypertension** (%)	58 (39.2)	12 (50)	46 (37.1)	0.24
**Metabolic syndrome** (%)	42 (28.4)	9 (37.5)	33 (26.6)	0.28
**Albumin** (g/L)	41.7+3.9	39.2+6.5	42.1+3	0.03
**Bilirubin** (μmol/L)	17.6+11.4	23.6+20.4	16.2+7.7	0.03
**Total cholesterol** (mmol/L)	4.8+1.2	4.4+1.1	4.9+1.2	0.1
**Glucose** (mmol/L)	6.8+3.1	8.8+5	6.3+2.2	0.002
**Fibrosis stage** (%)				
0	23 (15.5)	0	23 (18.5)	<0.001
1	53 (35.8)	1 (4.2)	52 (41.9)	
2	22 (14.9)	3 (12.5)	19 (15.3)	
3	28 (18.9)	8 (33.3)	20 (16.1)	
4	22 (14.9)	12 (50)	10 (8.2)	
**Steatosis grade** (%)				
1	22 (14.9)	5 (20.8)	17 (13.7)	0.70
2	78 (52.7)	12 (50)	66 (53.2)	
3	48 (32.4)	7 (29.2)	41 (33.1)	
**HVPG** (mmHg)	6.1+4.9	9.9+4.3	5.4+4.7	<0.001
**APRI**	0.8+0.6	1.5+0.6	0.7+0.5	<0.001
**FIB-4**	1.9+1.6	3.6+2.2	1.5+1.2	<0.001
**NAFLD fibrosis score**	-1.7+2.1	0.1+2.3	-2+1.9	<0.001
**HSI**	39+5.9	37.4+5	39.2+6.1	0.3
**Ultrasound—severe steatosis** (%)	24 (16.2)	3 (12.5)	21 (16.9)	0.59
**Xenon-133 scan—severe steatosis** (%)	35 (23.6)	5 (20.8)	30 (24.2)	0.72

Legend: Results given as mean ± SD or n (%). BMI, body mass index. IU, international units; ALT, alanine aminotransferase; AST, aspartate aminotransferase; γGT, gamma-glutamyl transpeptidase; Hb1Ac, hemoglobin glycosylated; HVPG, hepatic venous pressure gradient; APRI, AST-to-Platelet Ratio; NAFLD, nonalcoholic fatty liver disease; HSI, hepatic steatosis index. The p-values refer to T-test or chi-square test between patients with clinical outcomes and those without clinical outcomes.

### Incidence of clinical outcomes

**Fig 2:**
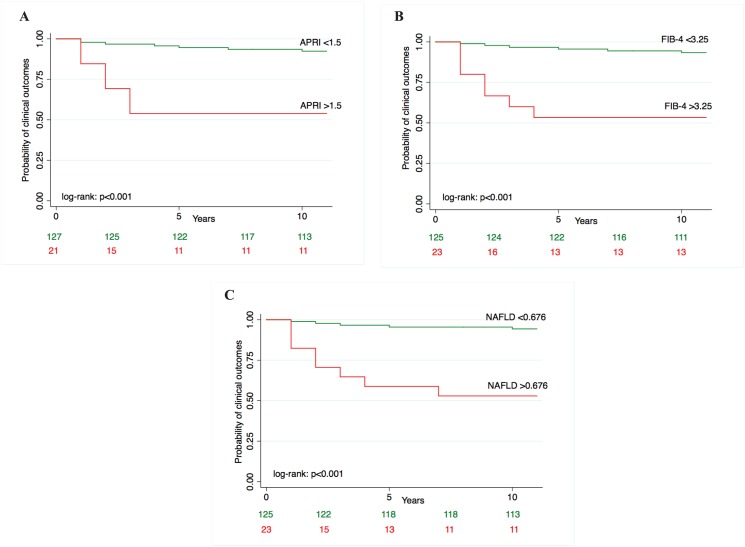
Survival curves of probability of clinical outcomes by: (A) APRI fibrosis category; (B) FIB-4 fibrosis category; (C) NAFLD fibrosis score category.

**Fig 3:**
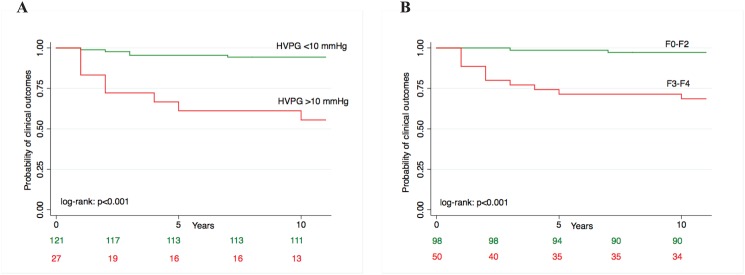
Survival curves of probability of clinical outcomes by: (A) HVPG category; (B) histologic fibrosis category.

Over a median follow-up of 5 years (IQR 3–8), 24 (16.2%) patients developed clinical outcomes, 11 (7.4%) died or underwent liver transplantation. There were 13 cases of ascites, 10 cases of de novo esophageal varices or bleeding and 1 case of HCC. Causes of death included cardiovascular disease in 4 cases, liver related in 3 cases, endocrinologic in 2 cases. 2 patients underwent liver transplantation. The overall incidence rate was 1.2/100 PY (95% CI 0.7–2.1). Incidence rates of clinical outcomes by histologic fibrosis risk category, HVPG risk category and serum fibrosis biomarkers risk category are depicted in Table 2. Survival curves of development of clinical outcomes by fibrosis risk category of histologic fibrosis, HVPG, APRI, FIB-4 and NAFLD fibrosis score and relative log-rank tests are reported in Fig 2 and S1. Incidence of clinical outcomes was significantly higher in patients in the high risk category by APRI (Fig 2A), FIB-4 (Fig 2B), NAFLD fibrosis score (Fig 2C), HVPG (Fig 3A) and histologic fibrosis (Fig 3B).

**Table 2:** Incidence rates of clinical outcomes according to fibrosis and risk category defined by non-invasive tools, liver histology and HVPG.

	Incidence rate per 100 PY (95% CI)
**Histologic fibrosis stage**	
Stage 3–4	3.7 (2–6.7)
Stage 0–2	0.2 (0.06–1)
**HVPG**	
>10 mmHg	5.9 (2.9–11.7)
< 10 mmHg	0.5 (0.2–1.3)
**APRI**	
>1.5	6.7 (3–15)
<1.5	0.7 (0.3–1.5)
**FIB-4**	
>3.25	6.9 (3.2–14.4)
<3.25	0.6 (0.2–1.4)
**NAFLD fibrosis score**	
>0.676	6.7 (3.3–13.3)
<0.676	0.5 (0.2–1.3)

Legend: PY, persons year; CI, confidence interval; HVPG, hepatic venous pressure gradient; APRI, AST-to-Platelet Ratio; NAFLD, nonalcoholic fatty liver disease.

### Predictors of development of clinical outcomes by multivariate analysis

The results of the multivariate Cox regression analysis of predictors of clinical outcomes are shown in Table 3. After adjustment for age, sex, diabetes, the serum fibrosis biomarkers APRI, FIB-4 and NAFLD fibrosis score, as well as HVPG and advanced fibrosis stage, were significantly associated with clinical outcomes developed during the follow-up period. Neither histologic steatosis and NAS, nor non-invasive steatosis methods predicted outcomes (data not shown).

**Table 3:** Multivariate hazard ratios of clinical outcomes by high-risk fibrosis category.

Variable	Unadjusted HR (95% CI)	Adjusted HR (95% CI)	p
**APRI >1.5** ^1^	7.84 (2.62–23.5)	5.02 (1.6–15.7)	0.006
**FIB-4 >3.25** ^2^	9.26 (3.1–27.75)	6.33 (1.98–20.2)	0.002
**NAFLD fibrosis score >0.676** ^3^	10.6 (3.45–32.6)	11.9 (3.79–37.4)	<0.001
**Histologic fibrosis stage F3-F4** ^4^	3.61 (1.70–7.67)	3.14 (1.41–6.95)	0.005
**HVPG >10 mmHg** ^5^	9.60 (3.13–29.44)	9.60 (3.07–30.12)	<0.001

^1^ Adjusted by age, sex, diabetes

^2^ Adjusted by sex, diabetes

^3^ Adjusted by sex

^4^ Adjusted by age, sex, diabetes

^5^ Adjusted by age, sex, diabetes

Legend: HR, hazard ratio; CI, confidence interval; HVPG, hepatic venous pressure gradient; APRI, AST-to-Platelet Ratio; NAFLD, nonalcoholic fatty liver disease. HR and relative p-values were estimated by Cox proportional hazard regression analysis.

### Performance of histology, HVPG and non-invasive fibrosis tools to predict clinical outcomes

**Fig 4:**
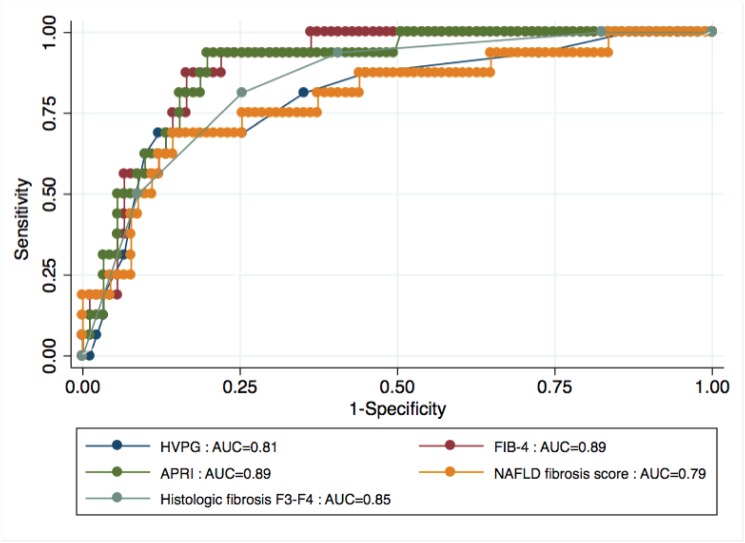
AUC of HVPG, histologic fibrosis stage, APRI, FIB-4 and NAFLD fibrosis score for prediction of clinical outcomes.

The prognostic accuracy of fibrosis stage, HVPG and serum fibrosis biomarkers is described in Table 4. Overall, serum fibrosis biomarkers had a good performance to predict clinical outcomes. There was no statistical difference in prognostic performance between serum biomarkers and histologic fibrosis stage or HVPG (p >0.05; Fig 4). Both histologic steatosis grade and non-invasive steatosis methods had poor performance in predicting clinical outcomes (AUC <0.50). Transient elastography was available in 45 patients. The prognostic accuracy of transient elastography in this subgroup of patients was also good, with an AUC of 0.87 (95% CI, 0.77–0.97). In most cases, transient elastography examination was too far away from liver biopsy to allow a direct prognostic comparison with histology and HVPG. We also explored if the combination of different non-invasive tests could increase the prognostic accuracy. There was no gain in prognostic accuracy by the combination of FIB-4 and NAFLD fibrosis score (data not shown). An alternative combination approach would have been to combine FIB-4 and or NAFLD/fibrosis score with transient elastography, given their inter-independence. However, given that transient elastography was available only for a subgroup of patients, we were not able to model a combination algorithm with it.

**Table 4:** AUC, accuracy, sensitivity, specificity, predictive values, likelihood ratios of baseline liver histology, HVPG and non-invasive fibrosis biomarkers to predict clinical outcomes.

	Fibrosis stage	HVPG	APRI	FIB-4	NAFLD fibrosis score
**AUC**±**SE (95% CI)**	0.85±0.04 (0.76–0.93)	0.81±0.06 (0.70–0.91)	0.89±0.03 (0.82–0.96)	0.89±0.03 (0.83–0.95)	0.79±0.06 (0.69–0.91)
**Accuracy (%)**	75.9	86.1	86	86	84.1
**Sensitivity**	81.3	62.5	50	56.3	50
**Specificity**	75	90.2	92.3	91.2	90.1
**PPV**	36.1	52.6	50	52.9	47
**NPV**	95.8	93.3	92.3	92.2	91.1
**LR+**	3.25	6.4	6.5	6.4	5.06
**LR-**	0.25	0.42	0.54	0.48	0.55

Legend: HVPG, hepatic venous pressure gradient; APRI, AST-to-Platelet Ratio; NAFLD, nonalcoholic fatty liver; AUC, area under the curve; SE, standard error; CI, confidence interval; PPV, positive predictive value; NPV, negative predictive value; LR, likelihood ratio. Accuracy, sensitivity, specificity, PPV, NPV and LR are computed based on the cut-off values adopted in the multivariate analysis: histologic fibrosis F3-F4, HVPG>10, APRI≥1.5, FIB-4>3.25, NAFLD fibrosis score>0.676.

## Discussion

This longitudinal study shows that serum fibrosis biomarkers predict clinical outcomes in patients with NASH. To our knowledge, this is the first study dissecting the prognostic value of non-invasive fibrosis and steatosis tools in patients with NASH as compared to both HVPG and histologic fibrosis stage. Our findings on prognostic value of simple fibrosis biomarkers are similar to those reported by Angulo et al in patients with NAFLD [25]. However, differently from that study, we specifically included only patients with NASH. One of the major strengths of our study is that we compared the prognostic performance of serum fibrosis biomarkers with both histologic fibrosis stage and HVPG. We found that the prognostic accuracy of fibrosis biomarkers is similar to that of these invasive reference methods for liver fibrosis and portal hypertension diagnosis. HVPG is a clinically meaningful reference standard for prognosis because portal hypertension constitutes the pathophysiological basis of cirrhosis complications. Esophageal varices, ascites and other end-stage complications of liver cirrhosis invariably occur in those with clinically significant portal hypertension defined as HVPG >10mmHg [8]. However measurement of HVPG and liver histology are unfeasible for serial monitoring because of invasiveness, cost, waiting times at radiologic facilities and lack of a standard therapeutic intervention for NASH. In tertiary care settings, these readily available and economic fibrosis biomarkers may potentially reduce the number of transjugular liver biopsies with HVPG measurement performed for prognostication. In primary and secondary care facilities, simple serum biomarkers can help targeting patients with an established diagnosis of NASH who need expedited referral to tertiary care centers. In line with previous histologic and imaging studies, we also showed that non-invasive diagnostic tools for hepatic steatosis lack predictive value for clinical outcomes in patients with NASH [10,32,33].

NASH is the evolutive counterpart of NAFLD and the most frequent liver disease in Western countries. NASH represents the second or third leading indication for liver transplantation in North America and it is projected to become the leading indication in the next 10–20 years [5,6]. This will inevitably change the physiognomy of liver transplant waiting lists and impact on organ supply [34]. Currently, there is no standard treatment for NASH. Apart for life style modifications, which are seldom implemented by the patients, there are few therapeutic alternatives. Vitamin E has been recommended as first line pharmacotherapy in patients with biopsy-proven NASH [1]. However, due to the lack of specific data, vitamin E is not recommended in diabetics and those with cirrhosis, which represent a significant proportion of patients with NASH. Moreover, concerns about potential risk for increase of all-cause mortality and for higher incidence prostate cancer limit the implementation of this treatment [35,36]. Weight loss induced by bariatric surgery may improve insulin resistance, liver fibrosis, steatosis and inflammation, although well-designed, randomized controlled studies are lacking [37]. Long wait times have been reported and this was associated with short-term risk of death for patients awaiting bariatric surgery [38]. Other therapeutic approaches have been proposed, but none of them have been deemed sufficiently safe or effective [1]. As such, serial monitoring strategies of NASH patients aimed at early identification of those at higher risk for baleful prognosis can help target interventions with specialized healthcare personnel, including serial dietetic counseling and monitoring of weight reduction by a nutritionist, and regular follow-up with an endocrinologist for optimal control of dysmetabolisms. Due to the epidemics of obesity and diabetes, these specialized resources are frequently saturated. Simple prognostic tools usable for serial monitoring may help the physician to dispose and optimize local resources more efficiently. Patients in high-risk fibrosis category may have a more favorable risk benefit ratio for vitamin E treatment and could be prioritized for bariatric surgery. The worse prognosis of patients in high-risk fibrosis category may also act as a stronger argument for patients’ compliance towards recommended life style modification. Moreover, given the limited organ pool in liver transplant list, simple serum fibrosis biomarkers may help individualize high-risk patients for liver transplant counseling and expedited referral.

During a median follow-up period of 5 years, 16% of our study population developed clinical outcomes. Those who developed clinical outcomes were more frequently diabetic, had lower platelets and albumin, higher bilirubin, fibrosis stage and HVPG at baseline. Bilirubin is incorporated in the Model for End-Stage Liver Disease (MELD) score. We did not employ MELD score to predict clinical outcomes in this study. Indeed, previous prognostic studies showed that MELD score is not useful to predict clinical outcomes when the study population does not include mostly cirrhotic patients [26]. Diabetes is a known risk factor for malignancy and fibrosis progression rate in chronic liver diseases of various etiologies [39]. Platelet count is an indirect marker of more advanced liver disease and portal hypertension [40]. Albumin is primarily synthesized by hepatocytes and is a sensitive marker of liver function. Dropping values indicate progressive liver impairment [41]. Because many of these variables are included in the fibrosis biomarkers we adopted, baseline APRI, FIB-4 and NAFLD fibrosis score were all significantly higher in patients who developed clinical outcomes during the follow-up period. On multivariate analysis, high-risk categories of both invasive (histologic fibrosis and HVPG) and non-invasive (APRI, FIB-4 and NAFLD fibrosis score) tools had significant HR for prediction of clinical outcomes. The prognostic performance of serum fibrosis biomarkers was similar to HVPG and histologic fibrosis stage.

Interestingly, neither histologic hepatic steatosis >66% nor non-invasive tools for steatosis diagnosis were significantly associated with clinical outcomes. Controversy surrounding this topic exists. Hepatic steatosis on ultrasonography was independently associated with cardiovascular disease in a population survey [12]. In a US community-based cohort study of 337 type 2 diabetic patients, a histologic or ultrasonographic diagnosis of NAFLD had a HR of 2.2 to predict overall mortality and NAFLD was associated with significantly higher mortality caused by liver disease and malignancy [13]. However, a study of 2342 type 2 diabetic patients concluded that steatosis >30% lacks predictive value for adverse clinical outcomes [33]. Our finding is in line with histologic paired biopsy studies which showed that steatosis alone is a weaker predictor for progressive NASH than liver fibrosis stage [32]. As suggested by Dunn et al, we hypothesize that loss of steatosis as NASH progresses and worsens over time may account for this finding [33]. In line with a recent report by Ekstedt and colleagues, we also found that NAS did not predict outcomes [10]. NAS is computed as the unweighted sum of the scores for steatosis, lobular inflammation and hepatocellular ballooning. Given that steatosis, graded on a 3-grade scale, does not have prognostic value, this could explain the lack of utility of NAS for prognostication.

This study has several strengths, including the availability of HVPG as reference standard for clinically significant portal hypertension and the long follow-up period. We acknowledge several limitations of this study. First, this study was conducted at a tertiary-care referral center, and although our prevalence of advanced liver fibrosis (33.8%), is comparable to other university centers, this is higher than that reported in primary/secondary care settings [40]. Second, this was a retrospective study, and as such we were unable to control for potential confounding factors, including the decision to proceed to transjugular liver biopsy, which could potentially select the sickest patients. Third, we did not have availability of more accurate non-invasive fibrosis methods, including transient elastography, Fibrotest-Fibrosure® and Enhanced Liver Fibrosis test. These non-invasive tools have shown high prognostic accuracy to predict outcomes in patients with chronic hepatitis C and B [24,26,42]. However, these tests are unlikely to be readily available in all clinical settings where NASH patients are managed. In a nationwide Canadian survey we have recently shown that difficult access to transient elastography can be a limiting factor for liver fibrosis screening, particularly in the setting of NAFLD [43].

In conclusion, this study shows that serum fibrosis biomarkers predict clinical outcomes in patients with NASH. The prognostic performance of simple serum biomarkers including APRI, FIB-4 and NAFLD fibrosis score is similar to that of HVPG and histologic fibrosis stage. In tertiary care settings, serum fibrosis biomarkers may potentially reduce the number of transjugular liver biopsies and HVPG measurement performed for risk stratification. In other clinical settings, they may help serial monitoring for prognostication of patients with an established diagnosis of NASH. Moreover, they might be employed for surveillance of progression or resolution of liver fibrosis in patients undergoing therapeutic interventions or enrolled in clinical trials of antifibrotic compounds.

Considering the epidemics of NASH in North America, the lack of a standard treatment and the future impact of this etiology of chronic liver disease on liver transplant waiting list, these simple tools may be used for targeting healthcare resources towards patients with potential worse prognosis, risk stratification, prioritization for vitamin E treatment and bariatric surgery, counseling and referral for liver transplantation.
